# Hematologic and molecular responses to ropeginterferon alfa‐2b therapy of polycythemia vera: 48‐week results from a prospective study

**DOI:** 10.1002/ijc.35411

**Published:** 2025-03-15

**Authors:** Seug Yun Yoon, Sung‐Soo Yoon, Deok‐Hwan Yang, Gyeong‐Won Lee, Sang Kyun Sohn, Ho‐Jin Shin, Sung Hwa Bae, Chul Won Choi, Eun‐Ji Choi, June‐Won Cheong, Soo‐Mee Bang, Joon Seong Park, Suk Joong Oh, Yong Park, Young Hoon Park, Sung‐Eun Lee

**Affiliations:** ^1^ Division of Hematology & Medical Oncology, Department of Internal Medicine Soonchunhyang University Seoul Hospital Seoul Korea; ^2^ Department of Internal Medicine Seoul National University College of Medicine Seoul Korea; ^3^ Department of Internal Medicine Chonnam National University Hwasun Hospital, Chonnam National University Medical School Jeollanam‐do Korea; ^4^ Division of Hematology‐Oncology, Department of Internal Medicine Institute of Health Science, Gyeongsang National University Hospital, Gyeongsang National University College of Medicine Jinju Korea; ^5^ Department of Hematology/Oncology Kyungpook National University Hospital Daegu Korea; ^6^ Department of Hematology/Oncology Pusan National University Hospital Busan Korea; ^7^ Division of Hematology/Oncology, Department of Internal Medicine Daegu Catholic, University Hospital, Daegu Catholic University School of Medicine Daegu Korea; ^8^ Division of Oncology and Hematology, Department of Internal Medicine Korea University Guro Hospital Seoul Korea; ^9^ Department of Hematology Asan Medical Center, University of Ulsan College of Medicine Seoul Korea; ^10^ Division of Hematology, Department of Internal Medicine Severance Hospital, Yonsei University College of Medicine Seoul Korea; ^11^ Department of Internal Medicine Seoul National University College of Medicine, Seoul National University Bundang Hospital Seongnam Korea; ^12^ Departments of Hematology‐Oncology Ajou University School of Medicine Suwon Korea; ^13^ Department of Oncology and Hematology Hanyang University Medical Center Seoul Seoul Korea; ^14^ Division of Hematology‐Oncology, Department of Internal Medicine Korea University College of Medicine Seoul Korea; ^15^ Division of Hematology‐Oncology, Department of Internal Medicine Ewha Womans University Mokdong Hospital Seoul Korea; ^16^ Department of Hematology Seoul St. Mary's Hospital, College of Medicine, The Catholic University of Korea Seoul Korea

**Keywords:** association, CHR, MR, polycythemia vera, ropeginterferon alfa‐2b

## Abstract

To prevent thrombosis in patients with polycythemia vera (PV), achieving a complete hematologic response (CHR) is highly recommended in practice. In addition, a reduced *JAK2* V617F mutation burden is expected to have a disease‐modifying effect, and its molecular response (MR) is currently of significant interest. This study aimed to assess the association between CHR and MR in patients with PV following treatment with ropeginterferon alfa‐2b. This phase 2, single‐arm, open‐label, investigator‐initiated trial was conducted at 16 sites in South Korea. Ninety‐nine patients were treated with ropeginterferon alfa‐2b subcutaneously every 2 weeks, at doses of 250 μg (week 1), 350 μg (week 3), and 500 μg (week 5), until week 48. CHRs were 27% (25/94), 46% (40/87), 56% (47/84), and 63% (51/81) at 12, 24, 36, and 48 weeks, respectively. The MR rates were 32% (28/88), 36% (29/81), 49% (38/77), and 57% (42/74) at 12, 24, 36, and 48 weeks, respectively. The Phi Coefficient for the association between CHR and MR was 0.6146 (*p* < .0001) at 48 weeks. In the subgroup analysis, patients with hydroxyurea resistance or intolerance, and those who were hydroxyurea‐naïve, had similar results in terms of the CHR. In conclusion, CHR and MR were observed to be associated in patients with PV treated with ropeginterferon.

## Graphical abstract

What's new?

Current treatment goals for polycythemia vera, a myeloproliferative neoplasm characterized by *JAK2* mutations, are mainly to prevent thrombosis. While ropeginterferon alfa‐2b treatment also reduces *JAK2* mutation burden, the correlation between the hematologic and molecular responses remains to be clarified. This single‐arm phase 2 study investigated the treatment response to ropeginterferon alfa‐2b as assessed by hematologic parameters and molecular markers and evaluated their association in patients with polycythemia vera requiring cytoreductive therapy. Patients who achieved a hematologic response exhibited a substantially reduced *JAK2* mutation burden compared to non‐responders. Molecular response was also associated with hydroxyurea‐naïve status and being female.

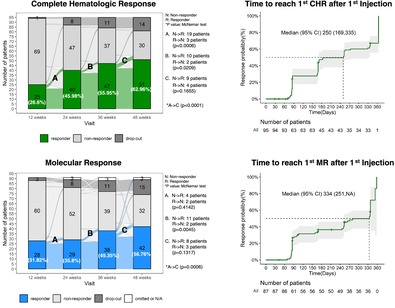

**Abbreviations**
CHR: complete hematologic response
ECOG: Eastern Cooperative Oncology Group
ELN: European LeukemiaNet
Hct: hematocrit
ICTRP: International Clinical Trials Registry Platform
IFNα: interferon‐α
MPNs: myeloproliferative neoplasms
MPN‐SAF TSS: MPN Symptoms Assessment Form Total Symptom Score
MR: molecular response
PV: polycythemia vera
SAE: serious adverse event
TEAE: treatment‐emergent adverse event
TRAE: treatment‐related adverse event
WBCs: white blood cells
WHO: World Health Organization

## 1 INTRODUCTION

Polycythemia vera (PV) is one of the most prevalent myeloproliferative neoplasms (MPNs), identified by the presence of activating somatic mutations in the *JAK2* gene and characterized by excessive production of red blood cells, platelets, and neutrophils. The *JAK2* Val617Phe point mutation was discovered in 2005.^1^ This point mutation replaces valine with phenylalanine at codon 617 of the *JAK2* gene and is present in 90% or more of patients with PV. It has a significant impact on the manifestation of clinical symptoms and disease progression. Current treatment goals for PV are to prevent thrombosis and manage symptoms. Phlebotomy, with a hematocrit (Hct) target of <45%, and daily low‐dose acetylsalicylic acid are considered standards of care for initial therapy regardless of disease risk.^2^, ^3^, ^4^ Ropeginterferon alfa‐2b is a recommended regimen for low‐risk patients, as well as high‐risk patients, who require cytoreductive therapy.^3^, ^5^

The mechanism of interferon‐α (IFNα) action remains unclear; however, IFNα has an apoptotic effect on *JAK2* V617F progenitor cells, leading to a significant and durable reduction in the *JAK2* mutation burden.^6^, ^7^, ^8^, ^9^, ^10^, ^11^ Increasing evidence suggests that IFNα treatment may have disease‐modifying potential,^12^ likely attributable to its ability to reduce *JAK2* mutation burdens, which are integral to the pathogenesis of PV.^13^, ^14^, ^15^ A meta‐analysis revealed a robust positive association between *JAK2* V617F allele burden and white blood cells (WBCs) and an increased risk of disease progression to myelofibrosis.^16^

Several studies with IFNα, including the ropeginterferon alfa‐2b, have reported an association between hematologic response and molecular response (MR).^17^, ^18^ However, the correlation between molecular and clinical response remains to be clarified. Therefore, this study investigated the hematologic response and MR in patients with PV requiring cytoreductive therapy with ropeginterferon alfa‐2b. We analyzed the associations between these responses and the safety and tolerability of ropeginterferon alfa‐2b.

## 2 METHODS

### 2.1 Study design

This phase 2, single‐arm, open‐label, investigator‐initiated trial was conducted at 16 sites in South Korea. The primary endpoint was the hematologic response and MR to ropeginterferon alfa‐2b and the association between the hematological response and MR. The study was designed to enroll 93 patients. The study was originally planned for 48 weeks but has been extended to 168 weeks (3 years) and is ongoing during the extension period. Further information regarding the study protocol, the clinical laboratory, the endpoints of the trial, the interruption of dosing, and additional results can be found in Appendix S1.

### 2.2 Inclusion and exclusion criteria

Patients who were diagnosed with PV according to the 2016 World Health Organization (WHO) criteria and requiring cytoreductive therapy, regardless of risk and previous treatment, were eligible if they were ≥19 years of age, were historically confirmed *JAK2* V617F positive, and had an elevated hematocrit of over 45% at screening. The exclusion criteria included contraindications to the use of any interferon and pregnant or lactating women. For a comprehensive list of inclusion and exclusion criteria, please refer to Appendix S1.

### 2.3 Treatment schedule

Patients were treated with ropeginterferon alfa‐2b, subcutaneously every 2 weeks, at a starting dose of 250 mcg, followed by 350 mcg at week 2, 500 mcg at week 4, and then until week 48 for the Core study period. Self‐injection was allowed, and patients were required to maintain a self‐injection diary to monitor compliance. Dose interruptions and reductions were permitted according to tolerability. Treatment was discontinued in cases of unresolved treatment‐related toxicity, loss of treatment efficacy, or withdrawal of consent. All patients received a low dose of acetylsalicylic acid during the study unless contraindicated. Other cytoreductive therapies were not permitted; however, the combination of phlebotomy with P1101 was allowed at the discretion of the investigator.

### 2.4 Patient evaluation

Patients were assessed every 12 weeks. Hematological parameters and chemistry were assessed in a local laboratory at each site. Quantitative *JAK2* V617F allele burden (*JAK2* Val617Phe Ipsogen® *JAK2* MutaQuant kit) was assessed centrally. The hydroxyurea resistance and intolerance criteria defined by modified European LeukemiaNet (ELN) criteria were applied in this study. Patient quality of life was evaluated using the Eastern Cooperative Oncology Group (ECOG) score and MPN Symptoms Assessment Form Total Symptom Score (MPN‐SAF TSS). Safety was assessed at each patient visit on the basis of reported adverse events, hematology, clinical chemistry, electrocardiography, and chest radiography.

### 2.5 Hematologic response criteria

A complete hematologic response (CHR) was defined by ELN criteria category B: durable (lasting at least 12 weeks) peripheral blood count remission, defined as hematocrit lower than 45% without phlebotomies; a platelet count ≤400 × 10^9^/L, WBC count <10 × 10^9^/L.

### 2.6 Molecular response criteria

MR was defined as a partial response on the basis of the 2009 ELN response criteria: (1) a reduction of ≥50% from the baseline value in patients with <50% mutant allele burden at baseline or (2) a reduction of ≥25% from the baseline value in patients with >50% mutant allele burden at baseline (applies only to a patient with a baseline value of mutant allele burden greater than 10%).

### 2.7 Statistical analyses

Primary analyses of the associations between MR and CHR at the last time point in the core study (48 weeks) were performed using the Chi‐squared test. Furthermore, the Phi coefficient was calculated to determine the degree of association between CHR and MR. Similarly, a comparison of decreased percentages of *JAK2* V617F in hematological responders and non‐responders was performed using a two‐sample *t*‐test or Wilcoxon's rank sum test at every assessment time point. Univariate and multivariate logistic regression analyses were performed to identify associations between clinical factors and MR. Variables with a *p* value <.05 in the univariable analysis were considered for multivariable analysis. Time‐to‐event outcomes as secondary endpoints were analyzed using Kaplan–Meier survival analysis. All efficacy endpoints used a full analysis set defined as subjects who received at least one dose of ropeginterferon alfa‐2b and met the inclusion/exclusion criteria with all data available for primary endpoint evaluation post‐baseline at least once. The safety endpoint was evaluated with a safety set defined as subjects who received at least one dose of ropeginterferon alfa‐2b. If available, the last visit data of patients who dropped out were included at the previous time point of the assessment visit. All analyses were performed using SAS software version 9.4 (SAS Institute, Cary, NC, USA) and R version 4.2.2 (R Foundation For Statistical Computing, Vienna, Austria).

## 3 RESULTS

### 3.1 Patient characteristics

The study started in October 2021, and the core treatment period was completed in November 2023. Ninety‐nine patients were enrolled, and 79 completed the core treatment (Figure S1). The median age of the patients was 58 years, with 51 males (54%) and 44 females (46%), 43 (45%) of whom were resistant and intolerant to hydroxyurea. There were 54 (57%) and 41 (43%) low and high‐risk patients, respectively. The median duration from diagnosis to enrollment was 32.4 months (range, 0.03–225.8) and the details of the baseline characteristics are described in Tables 1 and S4. According to the analysis set definition, 95 patients were included in the full analysis set, and 99 patients were included in the safety analysis set.

**TABLE 1:** Demographics and baseline characteristics.

	Total (*n* = 95)
Age, years, median (range)	58.0 (25–81)
Sex, no. (%)	
Female	44 (46.3)
Male	51 (53.7)
PV diagnosis, months, median (range)	32.43 (0.03–225.79)
Risk stratification, no. (%)	
Low	54 (56.8)
High	41 (43.2)
Hypertension, no. (%)	39 (41)
Diabetes, no. (%)	15 (16)
Aspirin use, no. (%)	75 (78.9)
Anticoagulant use, no. (%)	7 (7.4)
MPN‐SAF TSS score, median (range)	13.0 (0.0–60.0)
Hct (%), median (range)	49.6 (45.1–62.1)
Hgb (g/dL), median (range)	15.6 (12.0–21.0)
Platelets (10^9^/L), median (range)	545.0 (162–1772)
WBC (10^9^/L), median (range)	13.20 (4.91–48.57)
ANC (10^9^/L), median (range)	10.33 (2.67–42.26)
RBC (10^6^/L), median (range)	6.15 (4.23–8.87)
*JAK2* V617F mutation (%), range (*n* = 94^a^)	69.89 (0.44–97.17)

Abbreviations: ANC, absolute neutrophil count; Hct, hematocrit; Hgb, hemoglobin; HU, hydroxyurea; R/I, resistance or intolerance; RBC, red blood cell; WBC, white blood cell.

^a^ One patient baseline was omitted.

### 3.2 Complete hematologic response

**FIGURE 1:**
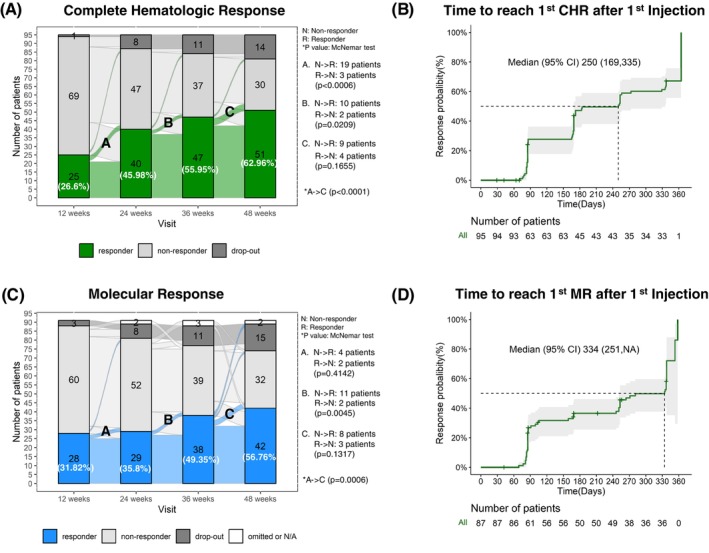
Complete hematology and molecular response. (A) Sankey‐style patient flow chart for Complete Hematology Response (CHR) rate by assessment visit. (B) Time to reach 1st CHR after the 1st injection of ropeginterferon alfa‐2b. (C) Sankey‐style patient flow chart for molecular response (MR) rate by assessment visit. (D) Time to reach 1st MR after the 1st injection of ropeginterferon alfa‐2b.

The CHR gradually increased during the treatment period and was 27% (25/94), 46% (40/87), 56% (47/84), and 63% (51/81) at 12, 24, 36, and 48 weeks, respectively (Figure 1A). The median time to reach the 1st CHR following the 1st injection was 250 days (95% CI: 169, 335) (Figure 1B). The average P1101 dose is presented in Table S2. The main reason for being assessed as non‐responders was an elevated hematocrit level. Other causes are listed in Table S1. Normalization of hematocrit levels took place after 24 weeks of treatment. Median and mean WBC and platelet counts were normalized after 12 weeks of treatment. Overall, the CHR rate increased steadily throughout the treatment period, regardless of subgroups (Figures S3–S5). In particular, a high CHR rate was observed in the hydroxyurea‐naïve group at all assessment visits; however, the difference between the two patient groups gradually decreased and was not significant at 48 weeks (71%, 32 out of 45; 53%, 19 out of 36, respectively; *p* = .0917) (Table S5).

### 3.3 Molecular response

**FIGURE 2:**
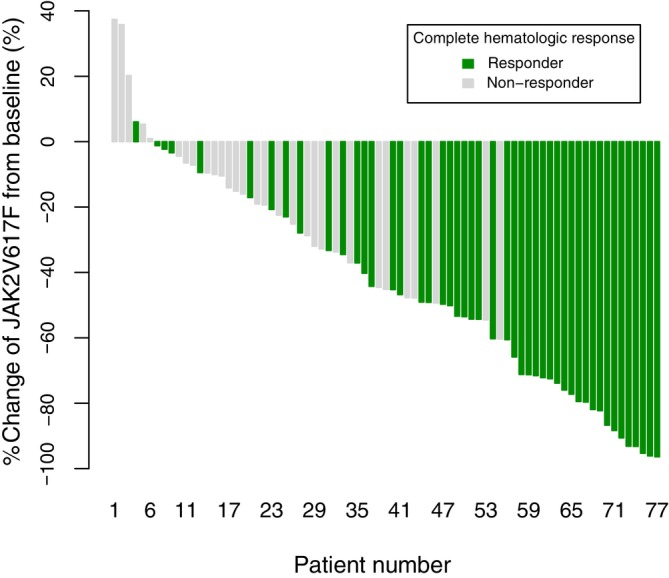
Waterfall plot of percentage of change from baseline in *JAK2* V617F allele burden in patients at 48 weeks based on response to CHR.

Since the evaluation of responders only applied to patients with a baseline value of *JAK2* V617F allele burden >10% as per the definition, the total number of evaluated patients differed between CHR and MR. MR showed an increasing trend similar to that of CHR, with 32% (28/88), 36% (29/81), 49% (38/77), and 57% (42/74) at 12, 24, 36, and 48 weeks, respectively (Figure 1C). The median time to achieve the 1st MR after the 1st injection was 334 days (95% CI: 251, NA) (Figure 1D). At 48 weeks, changes in the *JAK2* V617F allele burden from baseline were observed depending on whether CHR was achieved (Figure 2). Overall, a trend toward increasing MR and a steady decrease in mean and median absolute *JAK2* V617F allele burden were observed during treatment. In contrast, the reduction was slower in the hydroxyurea‐resistant or intolerant groups than that in the hydroxyurea‐naïve group (Table S6 and Figure S4).

### 3.4 Association between complete hematologic response and molecular response

A statistically significant association was observed between CHR and MR, with a particularly strong association at 48 weeks (Table 2). Furthermore, a greater percentage reduction in *JAK2* V617F allele burden was observed in CHR responders than in non‐responders at each assessment point (−57.40 ± 26.21 vs. −16.14 ± 23.51, *p* < .0001 in 48 weeks, CHR responders vs. non‐responders) (Table 3, Figure S2). In addition, when CHR was analyzed by its individual parameters, Hct, WBC, and Platelets, a significant association was observed between responders for each parameter and the achievement of MR at 48 weeks. In addition, the responder group had a substantial reduction in the *JAK2* V617F allele burden (Table 3).

**TABLE 2:** Factors including baseline clinical, hematologic characteristics and response at 48 weeks for association analysis by molecular response.

Parameters	All patients (*N* = 74)	Molecular response		*p* value
		Responder (*n* = 42)	Non‐responder (*n* = 32)	
Age, years, mean ± SD	55.2 ± 11.8	54.1 ± 13.1	56.7 ± 10.0	.355
Sex, no. (%)				
Male	40 (54.1%)	17 (40.5%)	23 (71.9%)	.014
Female	34 (45.9%)	25 (59.5%)	9 (28.1%)	
Disease risk, no. (%)				
Low	44 (59.5%)	27 (64.3%)	17 (53.1%)	.466
High	30 (40.5%)	15 (35.7%)	15 (46.9%)	
Hydroxyurea, no. (%)				
Naïve	41 (55.4%)	29 (69.0%)	12 (37.5%)	.014
R/I	33 (44.6%)	13 (31.0%)	20 (62.5%)	
Baseline Hct (%), median [Q1; Q3]	49.7 [47.2; 52.6]	50.2 [47.4; 54.0]	48.5 [46.9; 51.4]	.181
Baseline WBC (10^9^/L), median [Q1; Q3]	13.3 [10.7; 16.7]	13.6 [12.3; 16.9]	11.8 [9.5; 16.5]	.144
Baseline platelet (10^9^/L), median [Q1; Q3]	562.0 [425.0; 688.0]	562.0 [396.0; 682.0]	566.5 [437.5; 716.0]	.806
Baseline *JAK2* V617F mutation (%), mean ± SD	66.5 ± 20.5	71.5 ± 15.5	60.0 ± 24.3	.023
Baseline WBC >10 × 10^9^/L				
Yes	58 (78.4%)	35 (83.3%)	23 (71.9%)	.367
No	16 (21.6%)	7 (16.7%)	9 (28.1%)	
Baseline WBC >15 × 10^9^/L				
Yes	27 (36.5%)	16 (38.1%)	11 (34.4%)	.932
No	47 (63.5%)	26 (61.9%)	21 (65.6%)	
Baseline platelet >1000 × 10^9^/L				
Yes	5 (6.8%)	3 (7.1%)	2 (6.2%)	1.000
No	69 (93.2%)	39 (92.9%)	30 (90.8%)	
CHR, no. (%)	Phi coefficient 0.6146			
Yes	48 (64.9%)	38 (90.5%)	10 (31.2%)	.000
No	26 (35.1%)	4 (9.5%)	22 (68.8%)	
Hct responder, no. (%)	Phi coefficient 0.5396			
Yes	53 (71.6%)	39 (92.9%)	14 (43.8%)	.000
No	21 (28.4%)	3 (7.1%)	18 (56.2%)	
WBC responder, no. (%)	Phi coefficient 0.2739			
Yes	70 (94.6%)	42 (100.0%)	28 (87.5%)	.031
No	4 (5.4%)	0 (0.0%)	4 (12.5%)	
Platelet responder, no. (%)	Phi coefficient 0.2771			
Yes	67 (90.5%)	41 (97.6%)	26 (81.2%)	.038
No	7 (9.5%)	1 (2.4%)	6 (18.8%)	
Early CHR achievement, no. (%)				
Yes	18 (24.3%)	16 (38.1%)	2 (6.2%)	.004
No	56 (75.7%)	26 (61.9%)	30 (93.8%)	

*Note*: *p* value calculated *t*‐test or Mann–Whitney *U*‐test in continuous variables depend on Shapiro–Wilk test results. Chi‐square test was performed for *p* value in categorical variables, Fisher's exact test was performed if expected cell value <5.

**TABLE 3:** % change of *JAK2* V617F allele burden by CHR and each hematologic parameters responder versus non‐responder at 48 weeks.

	Mean ± SD (min, max) (*N* = 77)		
	Responder	Non‐responder	*p* value
CHR	−57.40 ± 26.21 (−96.36, 6.05)	−16.14 ± 23.51 (−54.27, 37.37)	<.0001
Hematocrit group	−53.06 ± 28.91 (−96.36, 6.05)	−17.14 ± 24.45 (−54.27, 37.37)	<.0001
WBC group	−44.47 ± 30.60 (−96.36, 37.37)	5.68 ± 22.78 (−19.35, 35.81)	.0019
Platelet group	−45.64 ± 29.93 (−96.36, 20.23)	−4.02 ± 30.79 (−44.59, 37.37)	.0008

*Note*: *p* value calculated by two‐sample *t*‐test.

The baseline characteristics of patients who achieved MR differed according to sex, subgroup related to hydroxyurea, and baseline *JAK2* V617F allele burden, but no other clinical or hematologic variables were significantly different. In particular, baseline *JAK2* V617F allele burden was significantly lower in MR non‐responders (60.0% ± 24.3%) than in responders (71.5% ± 15.5%) (*p* = .023). Achievement of CHR, hematocrit response, platelet response at 48 weeks, and early achievement of CHR at 12 weeks of treatment were significantly different between MR responders and non‐responders (Table 2).

We then performed univariable and multivariable analyses of factors potentially impacting the achievement of MR. Univariable analysis disclosed female gender (OR 3.76; 95% CI 1.44–10.5; *p* = .009), hydroxyurea naïve (OR 0.27; 95% CI 0.10–0.70; *p* = .008), CHR responder (OR 20.9; 95% CI 6.39–85.1; *p* < .001), hematocrit responder (OR 16.7; 95% CI 4.79–79.6; *p* < .001), platelet responder (OR 9.46; 95% CI 1.50–184; *p* = .043), CHR responder at 12 weeks (OR 9.23; 95% CI 2.34–61.9; *p* = .005), and baseline *JAK2* V617F allele burden (OR 1.03; 95% CI 1.01–1.06; *p* = .019) were significantly associated with MR. Female gender, hydroxyurea naïve, CHR responder, and CHR responder at 12 weeks were significantly associated with the achievement of MR in multivariable analysis. Hematocrit and platelet responders were excluded from the multivariable analysis model due to their multicollinearity with CHR responders (Table 4).

**TABLE 4:** Univariate and multivariable logistic regression analysis of factors associated with achievement of molecular response at 48 weeks.

Variables	Univariable		Multivariable	
	OR (95% CI)	*p* value	OR (95% CI)	*p* value
Female gender	3.76 (1.44–10.5)	.009	5.65 (1.35–29.9)	.025
HU R/I versus naïve	0.27 (0.10–0.70)	.008	0.19 (0.04–0.76)	.026
Baseline *JAK2* V617F mutation	1.03 (1.01–1.06)	.019	1.06 (1.02–1.11)	.009
Complete hematologic response at 48 weeks	20.9 (6.39–85.1)	<.001	26.5 (5.54–190)	<.001
Hematocrit responder at 48 weeks	16.7 (4.79–79.6)	<.001		
Platelet responder at 48 weeks	9.46 (1.50–184)	.043		
Early CHR achievement at 12 weeks	9.23 (2.34–61.9)	.005	4.61 (0.69–47.0)	.144

Abbreviations: CHR, complete hematologic response; CI, confidence interval; OR, odds ratio.

### 3.5 Safety and tolerability

In terms of safety, 192 treatment‐emergent adverse events (TEAEs) and 110 treatment‐related adverse events (TRAEs) were reported. In addition, 76% of patients experienced at least one TEAE, but most were mild or moderate in intensity. The most common TRAEs (%, *n*/99) were alopecia (13%, *n* = 13) and hepatobiliary‐related adverse events (35%, *n* = 35). The onset of TRAEs varied during the treatment period. TRAEs related to the hepatobiliary system and alopecia were frequently reported during the 85–168 days of treatment following the first injection of ropeginterferon alfa‐2b, whereas general disorders (e.g., Flu‐like syndrome, fatigue, and asthenia) were frequently reported during all time points of treatment. A total of 16 serious adverse events (SAEs) were reported, five of which were assessed as drug‐related adverse events (anemia, stable angina, hepatotoxicity, increased triglyceride levels, and bipolar disorder). No Grade 4 or 5 adverse events were observed. Treatment was discontinued in two patients due to TRAEs. Details of the adverse events are summarized in Table S3 and Figure S6. There were no deaths reported, and one patient reported disease progression during the core treatment period.

## 4 DISCUSSION

A previous study of ropeginterferon alfa‐2b, the PROUD/CONTINUATION trial, did not evaluate the correlation between molecular responses and outcomes.^13^, ^19^ In our study, we found an association between CHR and MR. Specifically, the group that achieved CHR during treatment had a reduced burden of the *JAK2* V617F allele compared with the group that did not achieve CHR. Furthermore, our findings suggest that, in addition to CHR, achievement of MR was also associated with the hydroxyurea‐naïve subgroup and female patients. These results are consistent with the findings from the PEGINVERA study.^18^ However, in a recent *JAK2* inhibitor study from Guglielmelli et al., the occurrence of a CCHR (complete clinical and hematologic response) was poorly correlated with the achievement of molecular responses and main clinical outcomes,^20^ which is in contrast to the results of our study. We assumed that the mechanism of action of interferon‐α may account for this difference. Ropeginterferon alfa‐2b is a PEGylated recombinant IFN‐α‐based agent that selectively inhibits malignant cells that drive neoplasms at the cellular level.^21^ Therefore, it effectively induces a durable response in hematologic and molecular parameters compared with other cytoreductive agents. Notably, the achievement of early CHR at 12 weeks of ropeginterferon alfa‐2b treatment was associated with a favorable MR, suggesting that this subgroup of patients may be particularly sensitive to interferon treatment, but further investigation is needed.

Our study has several limitations. Since the discovery of the *JAK2* V617F mutation, many studies have demonstrated that the achievement of an MR is associated with slower disease progression and event‐free survival.^13^, ^14^, ^20^, ^22^ In addition, a high *JAK2* V617F allele burden is a strong predictor of thrombosis and negative clinical outcomes,^20^ and these results suggest that reducing *JAK2* V617F is important for preventing thrombosis, which is one of the treatment goals of PV. Furthermore, other factors, such as spleen size, symptom burden, and additional mutations, are associated with progression and overall survival.^20^ However, the duration of our study was relatively short, and spleen size, bone marrow examination, or other mutations were not evaluated in this study. Owing to the short follow‐up period, it was not possible to definitively establish whether ropeginterferon alfa‐2b treatment prevents thrombosis or improves survival rates. An ongoing extension study is being conducted to further evaluate the long‐term efficacy and safety of ropeginterferon alfa 2b, as well as event‐free and overall survival, as a treatment option for PV. Moreover, some studies have suggested that achieving deep MR could potentially lead to disease modification in PV.^23^, ^24^ Despite the short follow‐up period in our study, the observed trend of decreasing *JAK2* V617F allele burden suggests the potential for deep or complete MR in the future.

The clinical meaning of *JAK2* V617F reduction and achievement of MR remains a subject of ongoing debate.^25^ Furthermore, the necessity of continuous monitoring of *JAK2* V617F allele burden throughout treatment, akin to hematologic parameters, is still contested and is not yet considered a mandatory parameter for routine monitoring. Despite this, our findings support the value of continuous monitoring of *JAK2* V617F allele burden, which has clinical benefits in patients with PV treated with ropeginterferon alfa‐2b.

In conclusion, an association between CHR and MR has been established, and ropeginterferon alfa 2b has demonstrated favorable clinical results for the treatment of patients with PV who require cytoreductive therapy.

## AUTHOR CONTRIBUTIONS

**Seug Yun Yoon:** Methodology; writing – original draft; investigation; formal analysis; visualization; validation. **Sung‐Soo Yoon:** Investigation; validation. **Deok‐Hwan Yang:** Investigation; validation. **Gyeong‐Won Lee:** Investigation; validation. **Sang Kyun Sohn:** Investigation; validation. **Ho‐Jin Shin:** Investigation; validation. **Sung Hwa Bae:** Investigation; validation. **Chul Won Choi:** Investigation; validation. **Eun‐Ji Choi:** Investigation; validation. **June‐Won Cheong:** Investigation; validation. **Soo‐Mee Bang:** Investigation; validation. **Joon Seong Park:** Investigation; validation. **Suk Joong Oh:** Investigation; validation. **Yong Park:** Investigation; validation. **Young Hoon Park:** Investigation; validation. **Sung‐Eun Lee:** Conceptualization; methodology; validation; writing – review and editing; funding acquisition; supervision.

## CONFLICT OF INTEREST STATEMENT

SYL's institution has received research funding from PharmaEssentia. The other authors declare no conflicts of interest.

## ETHICS STATEMENT

The trial was approved by the Ministry of Food and Drug Safety (clinical trial no. 33547) and registered in the International Clinical Trials Registry Platform (ICTRP, KCT0006138). Written informed consent was obtained from the patients, and the protocols were approved by the institutional review board or independent ethics committee at each site.

## Supporting information


**APPENDIX S1.** Supporting information.

## Data Availability

The data that support the findings of this study are available from the corresponding author upon reasonable request.
