# Supplementary material for: Hematologic and molecular responses to ropeginterferon alfa‐2b therapy of polycythemia vera: 48‐week results from a prospective study
Source: Int J Cancer. 2025 Mar 15;157(3):526–33. doi: 10.1002/ijc.35411 (PMC12141981; doi:10.1002/ijc.35411)
Supplement: Supplementary file 1 — APPENDIX S1. Supporting information. [file IJC-157-526-s001.pdf]

# Supplement Material

## Hematologic and molecular responses to ropeginterferon alfa-2b therapy of polycythemia vera: 48-week results from a prospective study

*Sung-Eun Lee, Seug Yun Yoon, Sung-Soo Yoon, Deok-Hwan Yang, Gyeong-Won Lee, Sang Kyun Sohn, Ho-Jin Shin, Sung Hwa Bae, Chul Won Choi, Eun-Ji Choi, June-Won Cheong, Soo-Mee Bang, Joon Seong Park, Suk Joong Oh, Yong Park, and Young Hoon Park*

### CONTENTS

1. Study scheme .......... 2
2. Inclusion and Exclusion criteria .......... 2
3. Clinical laboratory .......... 3
4. All endpoints in this clinical trial .......... 3
5. Dose interruption and reduction procedures .......... 6
6. Additional results .......... 6
7. Results of subgroup analysis (HU naïve vs HU Resistance or Intolerance / low-risk vs high-risk) .......... 10
   A. Baseline Characteristics .......... 10
   B. Complete hematologic response .......... 12
   C. Molecular response .......... 12

### LIST OF FIGURES

Figure S 1. Patient disposition .......... 13

Figure S 2. % change of JAK2V617F allele burden (%) by CHR .......... 14

Figure S 3. Change of hematologic (Hct, WBC, Platelets) parameters and JAK2V617F allele burden during the core treatment period .......... 15

Figure S 4. Change of hematologic (Hct, WBC, Platelets) parameters and JAK2V617F allele burden during the core treatment period by HU naïve vs R/I .......... 19

Figure S 5. Change of hematologic (Hct, WBC, Platelets) parameters and JAK2V617F allele burden during the core treatment period by Low-risk vs High-risk .......... 23

Figure S 6. Frequent drug-related AEs by treatment duration (according to investigators) .......... 26

### LIST OF TABLES

Table S 1. CHR and MR by assessment visit and detail of non-CHR at 48 weeks .......... 6

Table S 2. Average P1101 dose per injection by complete hematologic response and assessment visit .......... 7

Table S 3. Summary of Safety .......... 8

Table S 4. Baseline characteristics by subgroups .......... 10

Table S 5. Complete hematologic response by assessment visit .......... 12

Table S 6. Molecular response by assessment visit .......... 12

## 1. Study scheme

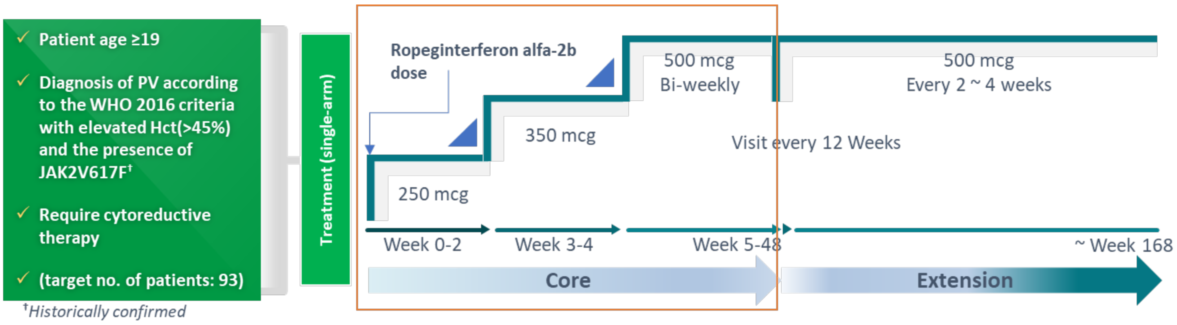


## 2. Inclusion and Exclusion criteria

- **Inclusion Criteria**

1. Male or female, 19 years or older

2. Subjects diagnosed with Polycythemia Vera and JAK2V617-positive according to the World Health Organization (WHO) 2016 criteria

3. Hematocrit level > 45% at screening

4. Patients who require cytoreduction treatment

5. Written informed consent obtained from the subject and ability for the subject to comply with the requirements of the Core study

- **Exclusion Criteria**

1. Any contraindications or hypersensitivity to pegylated interferon or any exposure to a non-pegylated or pegylated interferon alpha

2. Documented autoimmune disease at screening or in the medical history

3. Clinically relevant pulmonary infiltrates, pneumonia, and pneumonitis at screening that, in the Investigator's opinion, would jeopardize the safety of the subject or their compliance with the protocol

4. Infections with systemic manifestations (e.g., bacterial, fungal, or human immunodeficiency virus [HIV], except hepatitis B [HBV] and/or hepatitis C [HCV], at screening)

5. Known PV-related thromboembolic complications in the abdominal area (e.g. portal vein thrombosis, Budd-chiari syndrome) and/or splenectomy in the medical history

6. Use of any investigational drug <6 weeks prior to the first dose of study drug or not recovered from effects of prior administration of any investigational agent

7. History or presence of clinically relevant depression

8. Previous suicide attempts or at any risk of suicide at screening, in the judgment of the Investigator

9. Any significant morbidity or abnormality which may interfere with the study participation

10. Pregnant or lactating females or females who plan to become pregnant or women of childbearing potential and men who are not willing to use adequate contraceptive methods

*Adequate contraceptive methods: Barrier methods such as a condom and diaphragm; Intrauterine device (IUD); surgical methods; oral contraceptives; sexual abstinence, etc.

11. History of alcohol or drug abuse

12. Evidence of severe retinopathy (e.g., cytomegalovirus retinitis, macular degeneration) or clinically relevant ophthalmological disorder (due to diabetes mellitus or hypertension)

13. Thyroid dysfunction

14. History of major organ transplantation

15. History of uncontrolled severe seizure disorder

16. Leukocytopenia at the time of screening

17. Thrombocytopenia at the time of screening

18. History of any malignancy within 3 years (except Stage 0 chronic lymphocytic leukemia, basal cell, squamous cell, and superficial melanoma)

19. Subjects who are considered by the investigator to be ineligible for participation in this study

**3. Clinical laboratory**

- **Hematology:** HGB, HCT, RBC, Platelet, WBC (Absolute Neutrophil Count, Neutrophil, Lymphocyte, Monocyte, Eosinophil, Basophil)
- **Chemistry:** ALT, AST, GGT, ALP, Total bilirubin, Albumin, Total protein, Electrolytes (Potassium, Sodium), LDH, BUN, Creatinine, Glucose, Uric acid, LDL, HDL, Total cholesterol, Triglyceride

**4. All endpoints in this clinical trial**

- **Primary Efficacy Endpoint**

At each assessment visit after administration of the investigational product:

(1) A proportion of molecular[a] and hematological[b] responders after administration of the investigational product

(2) A reduction of JAK2 Val617Phe in hematological responders and non-responders

a) Definitions of Molecular Responders

Molecular response will be evaluated based on the Table below. For a reduction of any specific molecular abnormality to undetectable levels, it is evaluated as a complete response, and for a reduction of ≥ 50% from baseline value in patients with < 50% mutant allele burden at baseline, or a reduction of ≥ 25% from baseline value in patients with > 50% mutant allele burden at baseline, it is evaluated as a partial response. Complete response and partial response are evaluated as molecular responders, and other cases are evaluated as non-responders.

| Response Grade | Definition |
|---|---|
| Complete response | Reduction of any specific molecular abnormality to undetectable levels |
| Partial response* | • A reduction of ≥ 50% from baseline value in patients with < 50% mutant allele burden at baseline OR<br>• Reduction of ≥ 25% from baseline value in patients with > 50% mutant allele burden at baseline. |
| No response | Any response that does not satisfy partial response |

*Applies only to patients with a baseline value of mutant allele burden greater than 10%

b) Definitions of Hematological Responders

Hematological response will be evaluated based on the Table below. Hematologic responders are evaluated as a responders if it satisfies the category B, and a non-responders in other cases.

| | Criteria |
|---|---|
| **Complete remission** | |
| A | Durable* resolution of disease-related signs including palpable hepatosplenomegaly, large symptoms improvement† AND |
| B | Durable* peripheral blood count remission, defined as Ht lower than 45% without phlebotomies; platelet count ≤ $400x10^9$/L, WBC count < $10x10^9$/L, AND |
| C | Without progressive disease, and absence of any hemorrhagic or thrombotic event, AND |
| D | Bone marrow histological remission defined as the presence of age-adjusted normocellularity and disappearance of trilinear hyperplasia, and absence of > grade 1 reticulin fibrosis |
| **Partial remission** | |
| A | Durable* resolution of disease-related signs including palpable hepatosplenomegaly, large symptoms improvement† AND |
| B | Durable* peripheral blood count remission, defined as Hct lower than 45% without phlebotomies; platelet count ≤ $400x10^9$/L, WBC count < $10x10^9$/L, AND |
| C | Without progressive disease, and absence of any hemorrhagic or thrombotic event, AND |
| D | Without bone marrow histological remission defined as persistence of trilinear hyperplasia. |

| | Criteria |
|---|---|
| **No response** | Any response that does not satisfy partial remission |
| **Progressive disease** | Transformation into post-PV myelofibrosis, myelodysplastic syndrome or acute leukemia[‡] |

WBC, white blood cell.
[*]Lasting at least 12 wk.
[†]Large symptom improvement (≥ 10-point decrease) in MPN-SAF TSS.
[‡]For the diagnosis of post-PV myelofibrosis, see the IWG-MRT criteria; for the diagnosis of myelodysplastic syndrome and acute leukemia, see WHO criteria.

- **Secondary Efficacy Endpoints**

(1) Changes in the proportion of ELN response results for each category of A, B, C from baseline to each assessment visit after administration of the investigational product

(2) Time to first hematological response and duration of hematological response

The hematological response and duration of response are defined as follows.

- Hematological response: platelet ≤ 400x$10^9$/L & WBC < 10x$10^9$/L & HCT < 45%
- It is defined as the time from the start of the investigational product administration to the time of the documented hematological response.
- It is defined as the time from the first documented hematological response to the date when the criterion is no longer met.

- **Exploratory Endpoints**

(1) A proportion of subjects with ≥ 50% reduction in the MPN-SAF TSS total score from baseline to each assessment visit after administration of the investigational product, along with molecular response

(2) Changes in symptom scores using the MPN-SAF TSS by subject from baseline to each assessment visit after administration of the investigational product, along with molecular response

(3) Changes in JAK2 Val617Phe in subjects from baseline to each assessment visit after administration of the investigational product

(4) A number and percentage of subjects by injection schedule of extension study (PV ET)

(5) Changes in the proportion of existing ELN response category A and the proportion of modified ELN response category A[a] from baseline to each assessment visit after administration of the investigational product

a) Modified ELN response criteria A

Criteria A: Durable* resolution of disease-related signs including palpable hepatosplenomegaly and large symptoms improvement†

[*]Lasting at least 12 wk.

†Large symptom improvement in MPN-SAF TSS.

- Baseline TSS scores ≥32: 10-points reduction in TSS score
- Baseline TSS scores 18-<32, inclusive: 5-points reduction in TSS score
- Baseline TSS scores 8-<18, inclusive: TSS score decreases to ≤8
- Baseline TSS score <8: TSS score stays <8

The MPN-SAF TSS is a questionnaire to assess the symptoms of myeloproliferative neoplasm (MPN). MPN-SAF TSS items are scored from 0 (absent) to 10 (worst imaginable). MPN-SAF TSS results obtained 8 weeks prior to screening may be used for baseline, if available. Test results obtained within 10 days prior to each assessment visit may be used, if available.

## 5. Dose interruption and reduction procedures

Dose reduction or dose interruption of P1101 is recommended in subjects experiencing adverse events until the AE is resolved. Treatment should be discontinued and the End Of Study Visit should be completed if intolerability persists even after dose reduction or dose interruption. Any dose modification and the date and time of dose modification must be recorded in the eCRF.

The dose reduction of the investigational product should be driven only by the safety and tolerability of the drug under study. If a specific dose is not tolerated and drug-related toxicity occurs, the dose should be reduced to the next lower dose or interrupted according to the following scenario:

- If a subject experiences severe (Grade 3 or 4) toxicity or the absolute neutrophil count (ANC) falls below 0.5×109, treatment should be interrupted until recovery to allow continuation of treatment (e.g. Grade 1 and mild in intensity). Treatment should be resumed at a lower dose level than the one that led to the toxicity. For example, treatment should be resumed at 350 µg if a Grade 3 AE occurred at 500 µg. Dose re-escalation to the previous dose should be attempted if there is no response at the reduced dose after 3 months (platelet count > 600×109/L).
- If a subject experiences Grade 2 toxicity or ANC falls below 0.75×109/L but above 0.5×109/L, dose reduction should be considered without interruption of treatment.
- Grade 1 toxicity should not lead to dose reduction or dose interruption.

In subjects who have not recovered from previous toxicity, re-escalation of the dose may not be considered. The initial dose of P1101 is 250 µg. The highest dose of P1101 administered to subjects in this study should not exceed 500 µg every 2 weeks.

## 6. Additional results

**Table S 1. CHR and MR by assessment visit and detail of non-CHR at 48 weeks**

| %, (n/N) | CHR | MR |
|---|---|---|
| **12 weeks** | 26.6%, (25/94) | 31.82% (28/88) |
| **24 weeks** | 45.98% (40/87) | 35.80% (29/81) |

| %, (n/N) | CHR | MR |
|---|---|---|
| **36 weeks** | 55.95% (47/84) | 49.35% (38/77) |
| **48 weeks** | 62.96% (51/81) | 56.76% (42/74) |
| **Never achieved CHR until 48 weeks** | | 30.86%, (25/81) |
| **Loss of CHR at 48 weeks after achieving CHR during the core period** | | 6.17%, (5/81) |
| **Loss of hematocrit response at 48 weeks** | | 80%, (4/5) |
| **Loss of WBC response at 48 weeks** | | 20%, (1/5) |
| **Loss of platelet response at 48 weeks** | | 0%, (0/5) |

**Table S 2. Average P1101 dose per injection by complete hematologic response and assessment visit**

| Average dose/injection (µg) | | CHR | Non-CHR |
|---|---|---|---|
| **12 weeks** | Mean±SD | 430.33±15 | 426.09±27.08 |
| | Median (min, max) | 433.33 (358.33, 433.33) | 433.33 (300, 450) |
| **24 weeks** | Mean±SD | 463.88±73.32 | 479.26±57.18 |
| | Median (min, max) | 500 (250, 500) | 500 (225, 500) |
| **36 weeks** | Mean±SD | 428.19±95.64 | 473.65±66.65 |
| | Median (min, max) | 500 (250, 500) | 500 (200, 500) |
| **48 weeks** | Mean±SD | 419.12±99.2 | 462.22±88.73 |
| | Median (min, max) | 500 (250, 500) | 500 (200, 500) |

**Table S 3. Summary of Safety**

| Type of event | | Adverse events | Number of patients n(%), N = 99 |
|---|---|---|---|
| **Adverse events** | | 192 | 76 (76%) |
| **Serious adverse events** | | 16 | 16 (16%) |
| **Treatment-related serious adverse event** | | 5 | 5 (5%) |
| **Treatment-related adverse events** | | 110 | 56 (57%) |
| **Intensity of treatment-related adverse events** [a] | | | |
| | **Grade 1** | 66 | 43 (43%) |
| | **Grade 2** | 28 | 25 (25%) |
| | **Grade 3** | 12 | 7 (7%) |
| | **Grade 4,5** | 0 | 0 |
| **Treatment-related adverse events leading to discontinuation**[b] | | 2 | 2 (2%) |
| **Disease progression (myelofibrosis)**[c] | | 1 | 1 (1%) |
| **Thromboembolic adverse events**[d] | | 5 | 5 (5%) |
| **Deaths** | | 0 | 0 |
| **Events related to study treatment occurring in ≥3% of patients by PT**[a] | | | |
| **Anemia** | | 3 | 3 (3%) |

| Type of event | Adverse events | Number of patients n(%), N = 99 |
|---|---|---|
| **Alanine aminotransferase increased** | 14 | 14 (14%) |
| **Aspartate aminotransferase increased** | 10 | 10 (10%) |
| **Alopecia** | 13 | 13 (13%) |
| **Decreased appetite** | 3 | 3 (3%) |
| **Fatigue** | 4 | 4 (4%) |
| **Gamma-glutamyl transferase increased** | 9 | 9 (9%) |
| **Hepatic enzyme increased** | 4 | 4 (4%) |
| **Influenza-like illness** | 3 | 3 (3%) |
| **Liver function test abnormal** | 3 | 3 (3%) |
| **Myalgia** | 3 | 3 (3%) |
| **Platelet count decreased** | 8 | 8 (8%) |
| **Vomiting** | 3 | 3 (3%) |

a. Intensity grading according to CTCAE 5.0: Patients with multiple occurrences of the same adverse event were counted only once.
b. One patient experienced bipolar disorder and one patient experienced hepatotoxicity.
c. One patient developed myelofibrosis.
d. Cerebral artery stenosis, two cerebral infractions, angina pectoris, haematoma
*PT* preferred term

7. **Results of subgroup analysis (HU naïve vs HU Resistance or Intolerance / low-risk vs high-risk)**

A. **Baseline Characteristics**

**Table S 4. Baseline characteristics by subgroups**

| | HU naïve (n = 52) | HU R/I (n = 43) | Low-risk (n=54) | High-risk(n=41) |
|---|---|---|---|---|
| **Age, years, median (range)** | 56.5 (26–80) | 59.0 (37–81) | †50.00 (26-60) | 65 (53-81) |
| **Sex, No. (%)** | | | | |
| Female | 24 (46.1) | 20 (46.5) | 21 (38.9) | 23 (56.1) |
| Male | 28 (53.9) | 23 (53.5) | 33 (61.1) | 18 (43.9) |
| **PV diagnosis, months, median (range)** | 24.64 (0.04–169.61) | 73.96 (4.29–225.79) | 28.32 (0.04-225.79) | 56.57 (1.93-190.21) |
| **Risk stratification, No. (%)** | | | | |
| Low | 31 (59.6) | 23 (53.5) | N/A | |
| High | 21 (40.4) | 20 (46.5) | | |

| | HU naïve (n = 52) | HU R/I (n = 43) | Low-risk (n=54) | High-risk(n=41) |
|---|---|---|---|---|
| **Hypertension, No. (%)** | 21 (40) | 18(42) | 17 (31) | 22 (54) |
| **Diabetes, No. (%)** | 8 (15) | 7 (16) | 5 (9) | 10 (24) |
| **Hct (%), median (range)** | 50.00 (45.20–62.10) | 48.70 (45.10–60.40) | 48.70 (45.10-62.10) | 50.70 (45.20-61.70) |
| **Hgb (g/dl), median (range)** | 15.6 (12.7–21.0) | 15.8 (12.0–19.15) | 15.40 (12.60-21.00) | 15.90 (12.00-19.15) |
| **Platelets ($10^9$/L), median (range)** | 583(187–1772) | 485 (162–1062) | 597.50 (162-1772) | 464.00 (187-770) |
| **WBC ($10^9$/L), median (range)** | 13.31 (4.91–48.57) | 12.89 (5.96–34.50) | 12.44 (5.61-21.90) | 13.93 (4.91-48.57) |
| **ANC ($10^9$/L), median (range)** | 10.32 (2.67–42.26) | 10.58 (4.19–32.02) | 10.06 (3.44-19.37) | 11.32 (2.67-42.26) |
| **RBC ($10^6$/L), median (range)** | 6.57 (4.44–8.56) | 5.73 (8.87–4.23) | 6.28 (4.50-8.87) | 5.83 (4.23-8.30) |
| ***JAK2* V617F mutation (%), (range)** | [‡]68.71 (2.09–92.80) | 74.68 (0.44–97.17) | [‡]62.19 (0.44-94.06) | 80.77(2.09-97.17) |

**Abbreviations: HU, hydroxyurea; R/I, resistance or intolerance; Hct, Hematocrit; Hgb, Hemoglobin; RBC, Red Blood Cell; WBC, White Blood Cell; ANC, Absolute Neutrophil Count**

[†]4 patient baseline was omitted, [‡]1patient baseline was omitted.

### B. Complete hematologic response

**Table S 5. Complete hematologic response by assessment visit**

| %, (n/N) | HU naïve | HU R/I | P value | Low-risk | High-risk | P value |
|---|---|---|---|---|---|---|
| **12 weeks** | 41.18% (21/51) | 9.30% (4/43) | 0.0004 | 29.63% (16/54) | 22.5% (9/40) | 0.4446 |
| **24 weeks** | 59.57% (28/47) | 30.00% (12/40) | 0.0054 | 47.06% (24/51) | 44.44% (16/36) | 0.8122 |
| **36 weeks** | 65.22% (30/46) | 44.74% (17/38) | 0.0610 | 61.22% (30/49) | 48.57% (17/35) | 0.2547 |
| **48 weeks** | 71.11% (32/45) | 52.78% (19/36) | 0.0917 | 69.39% (34/49) | 53.13% (17/32) | 0.1419 |

### C. Molecular response

**Table S 6. Molecular response by assessment visit**

| %, (n/N) | HU naïve | HU R/I | P value | Low-risk | High-risk | P value |
|---|---|---|---|---|---|---|
| **12 weeks** | 45.65 % (21/46) | 16.67 % (7/42) | 0.0032 | 38.78% (19/49) | 23.08% (9/39) | 0.1189 |
| **24 weeks** | 48.84 % (21/43) | 21.05 % (8/38†) | 0.0088 | 47.83% (22/46†) | 20.00% (7/35) | 0.0093 |
| **36 weeks** | 61.90 % (22/42†) | 34.29 % (12/35†) | 0.0155 | 53.33% (24/45†) | 43.75% (14/32†) | 0.4138 |
| **48 weeks** | 70.73 % (29/41) | 39.39 % (13/33†) | 0.0064 | 61.36% (27/44) | 50.00% (15/30†) | 0.3394 |

†The sample has been excluded from the analysis due to DNA were lower than 5 ng/uL

## 8. Supplemental Figures

Figure S 1. Patient disposition

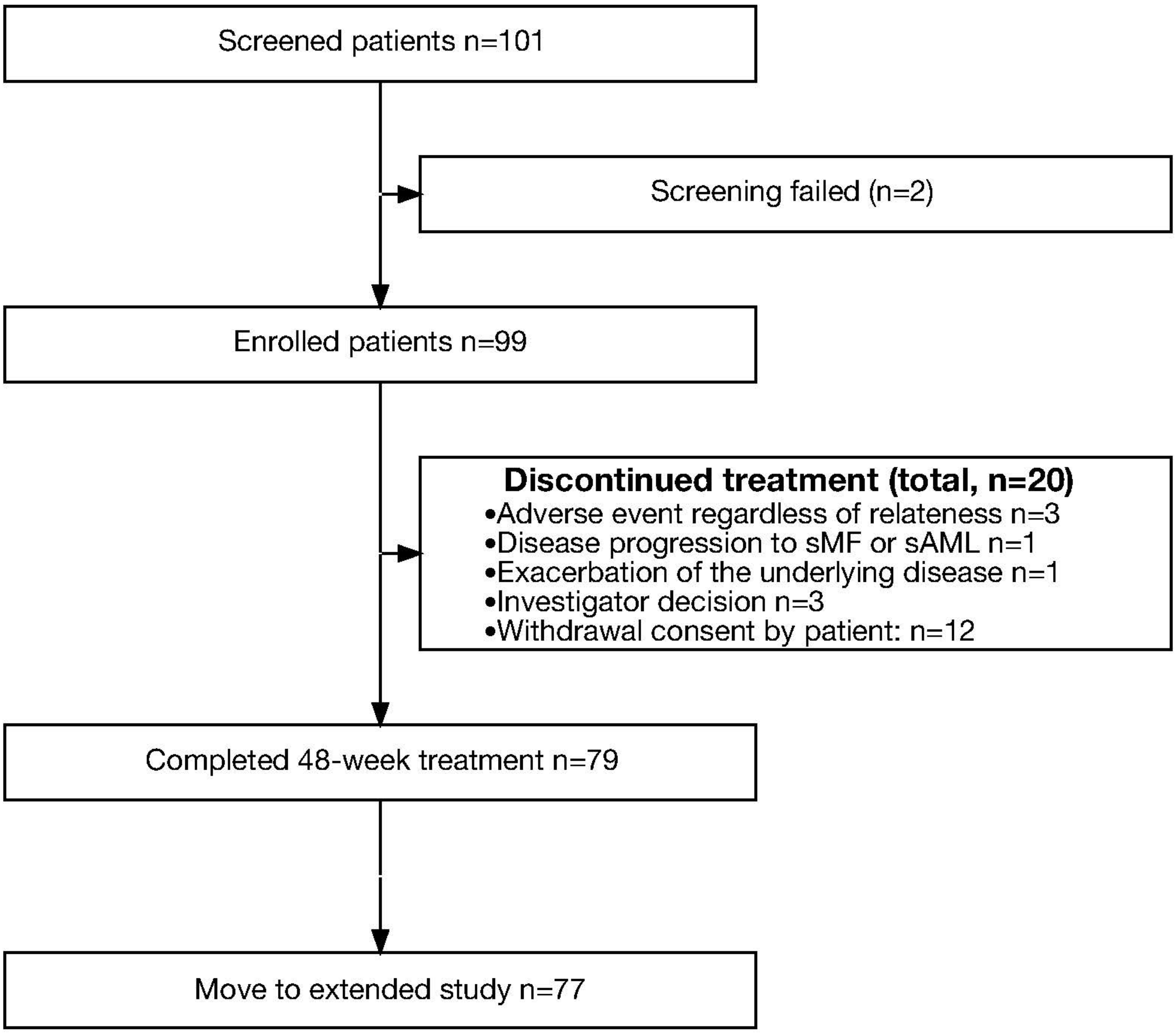

Figure S 2. % change of JAK2V617F allele burden (%) by CHR

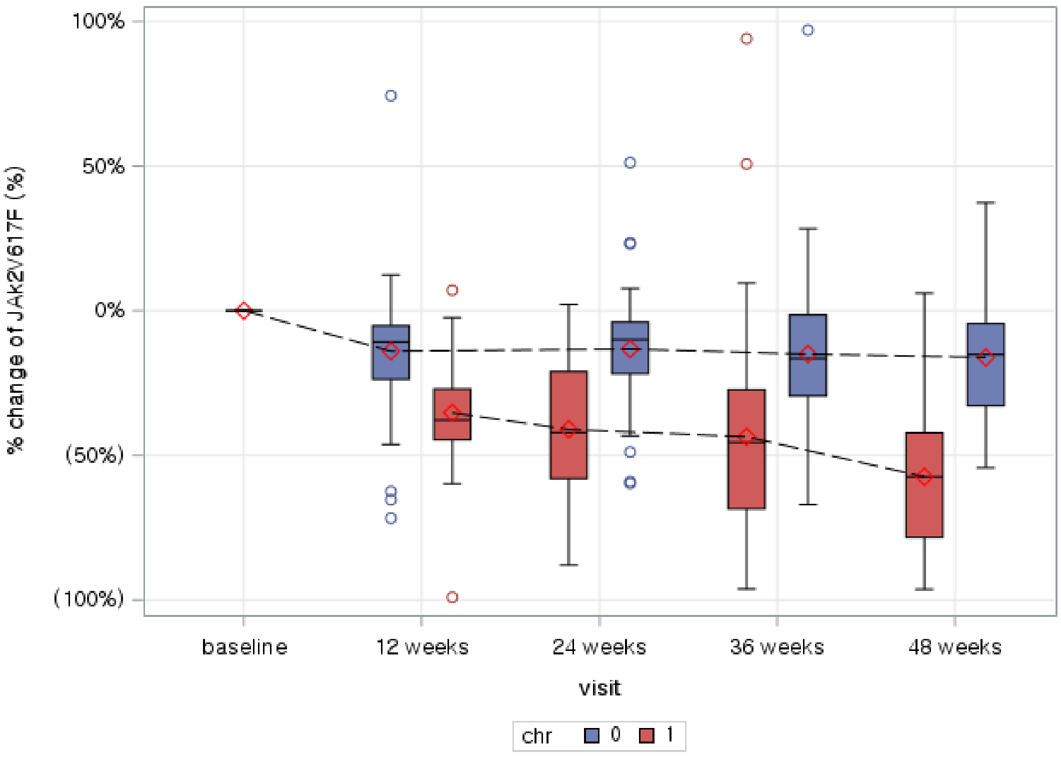


a. connect the means of the plot with straight lines

Figure S 3. Change of hematologic parameters ((A) Hct, (B) WBC, (C) Platelets) and (D) JAK2V617F allele burden during the core treatment period. The means of the plot are connected with straight lines, and the redline indicates baseline value for assessing CHR for each hematologic parameter (Hct 45%, WBC $10x10^9/L$, PLT $400x10^9/L$).

(A). Change of hematocrit (%)

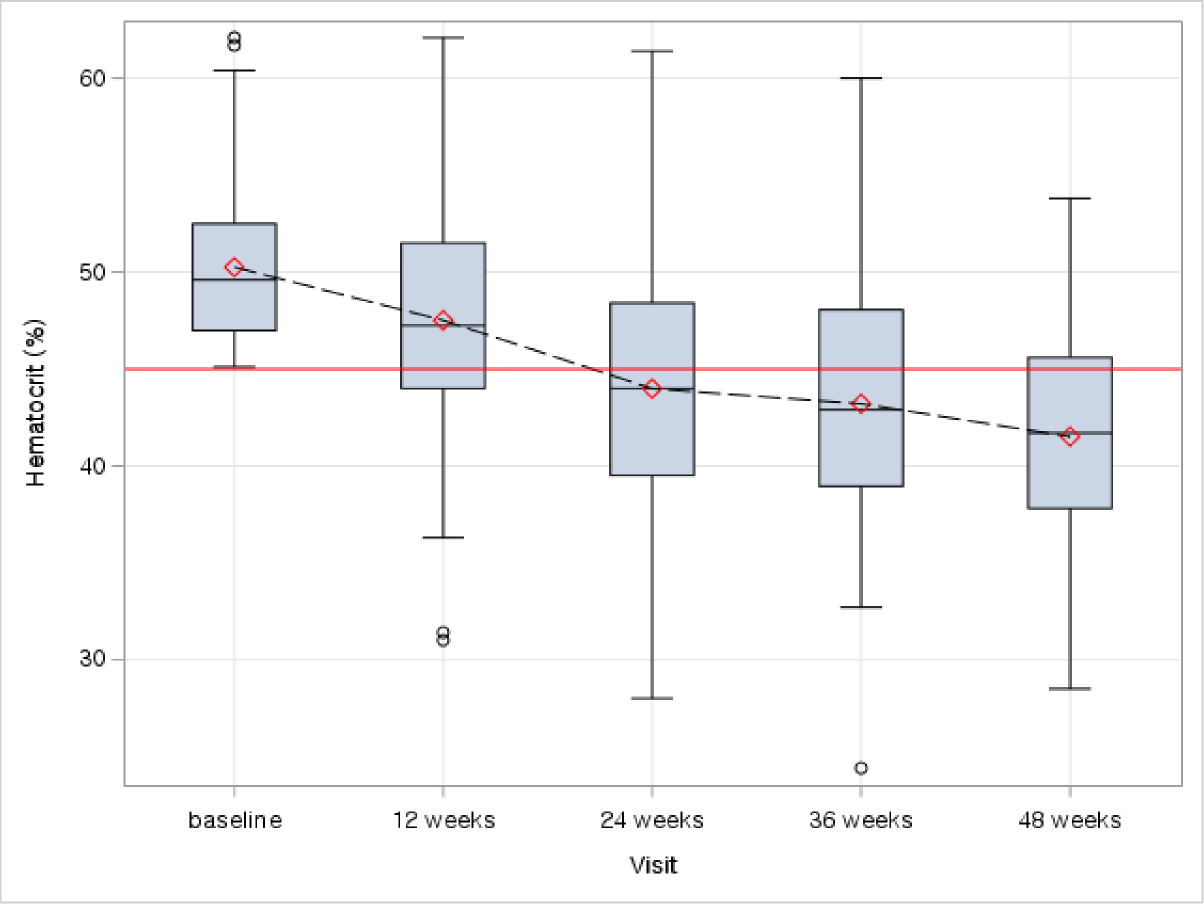

(B) Change white blood cell count ($10^9$/L)

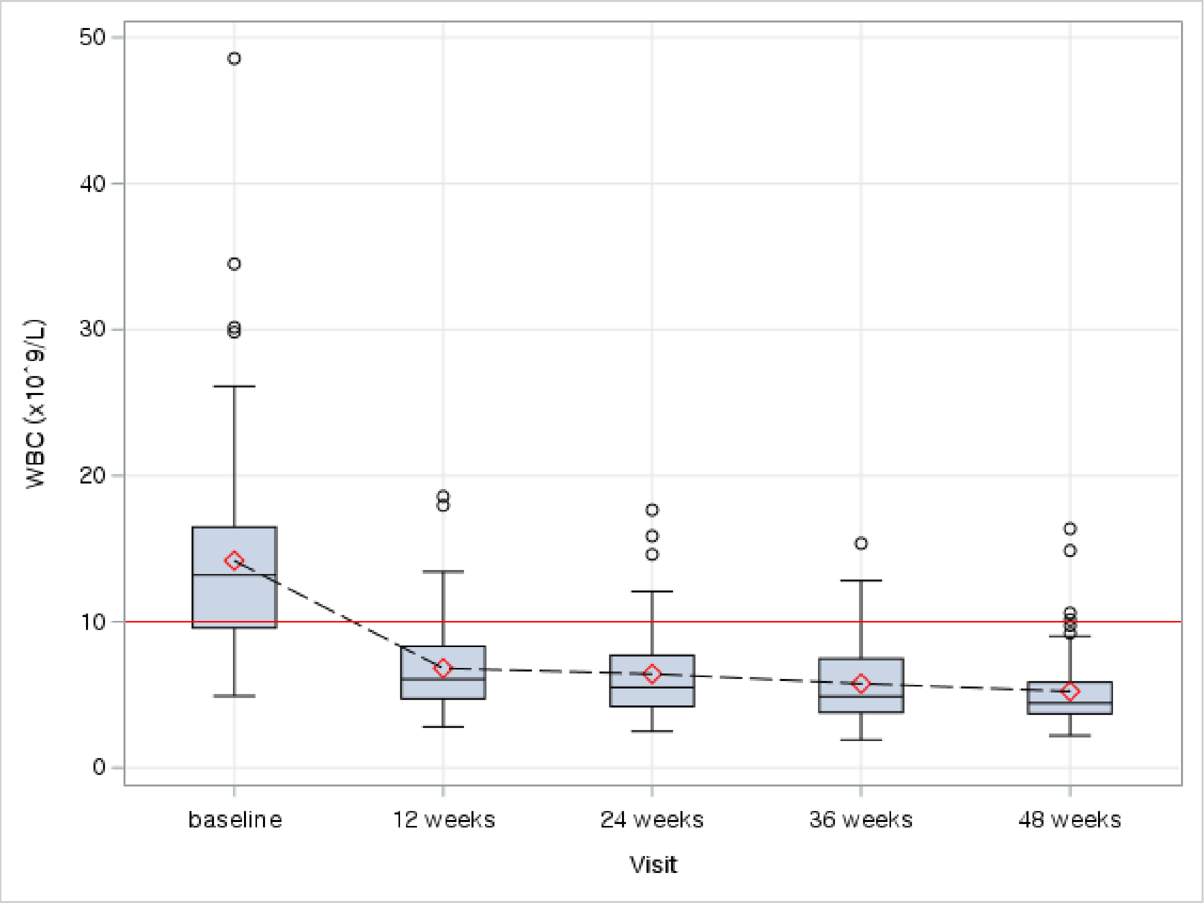

(C) Change platelet count ($10^9$/L)

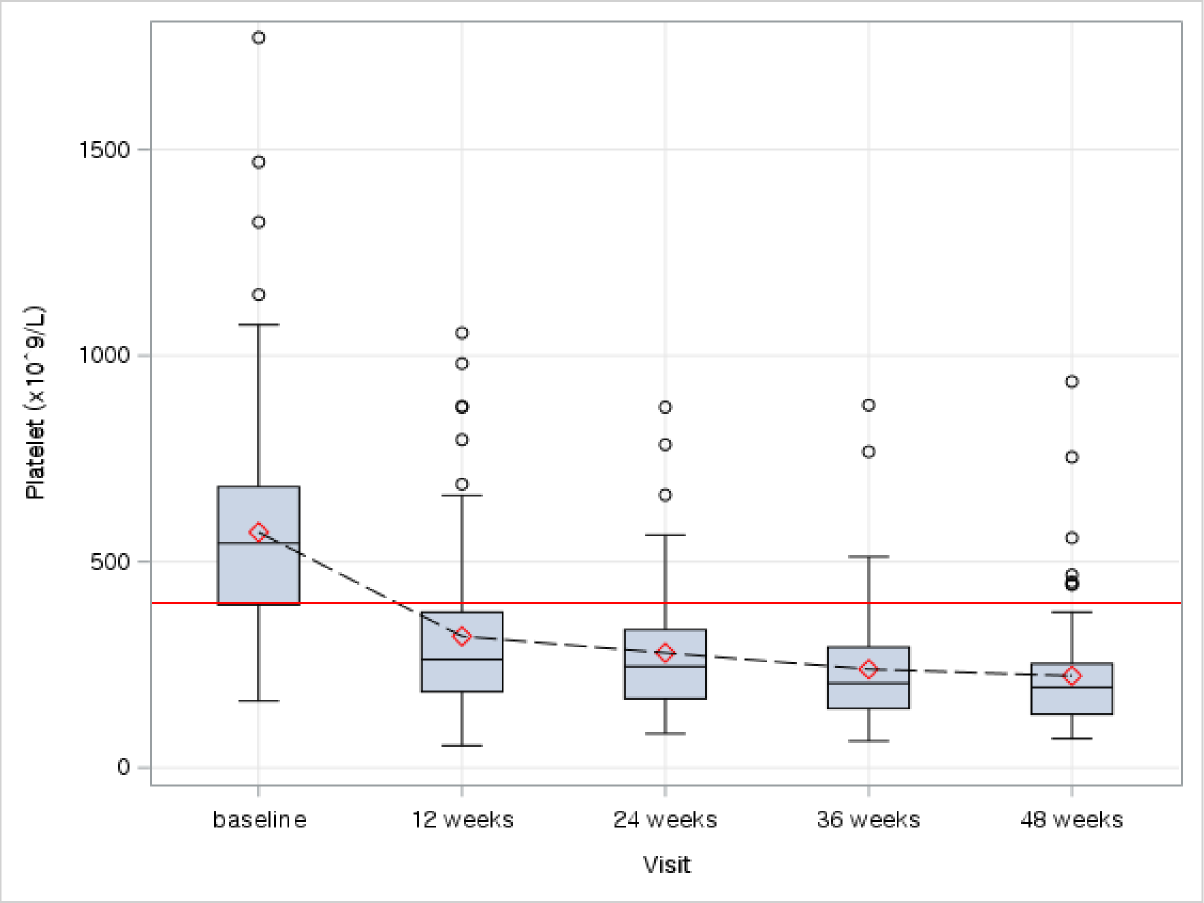

(D) % change of JAK2V617F allele burden (%)

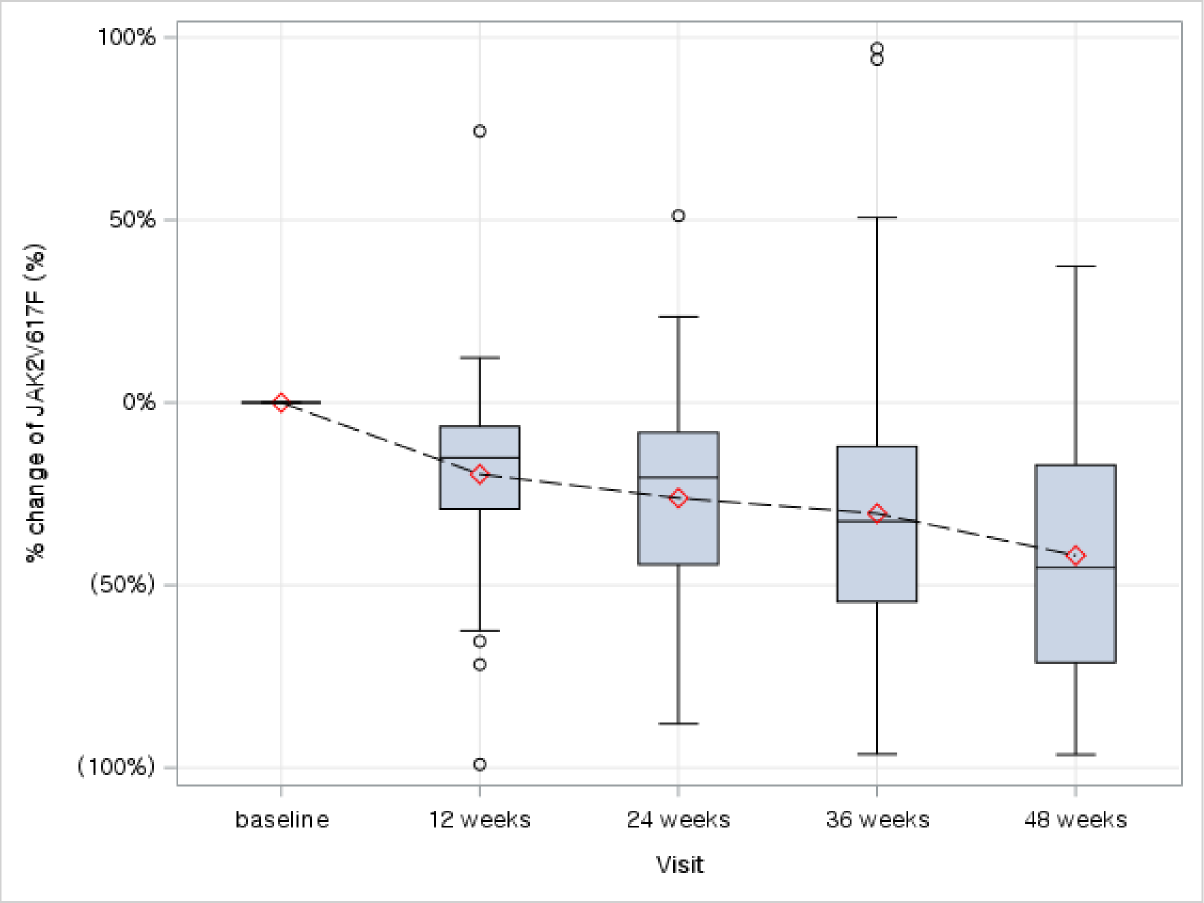

Figure S 4. Change of hematologic parameters ((A) Hct, (B) WBC, (C) Platelets) and (D) JAK2V617F allele burden during the core treatment period by HU naïve vs R/I. The means of the plot are connected with straight lines, and the redline indicates baseline value for assessing CHR for each hematologic parameter (Hct 45%, WBC $10x10^9/L$, PLT $400x10^9/L$).

(A) Change hematocirt (%)

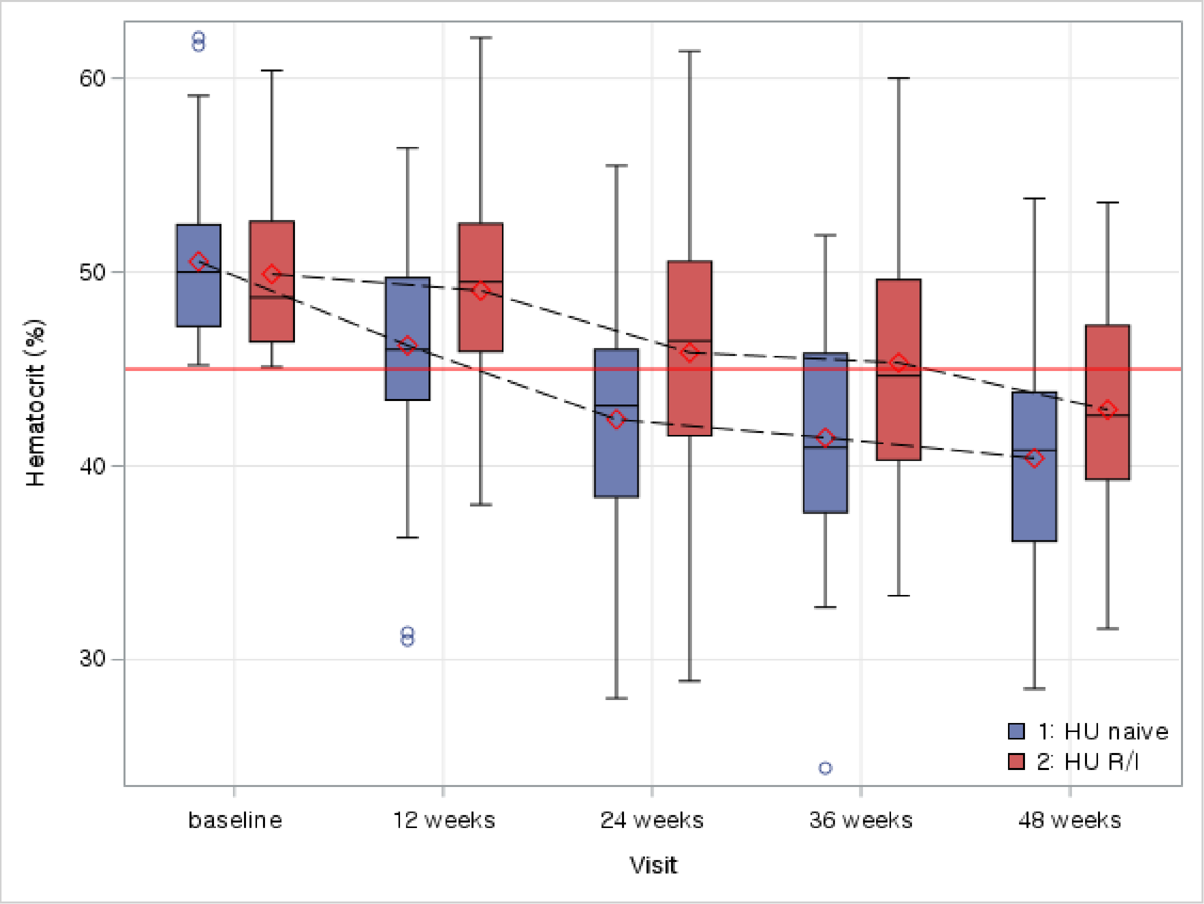

(B) Change white blood cell count ($10^9$/L)

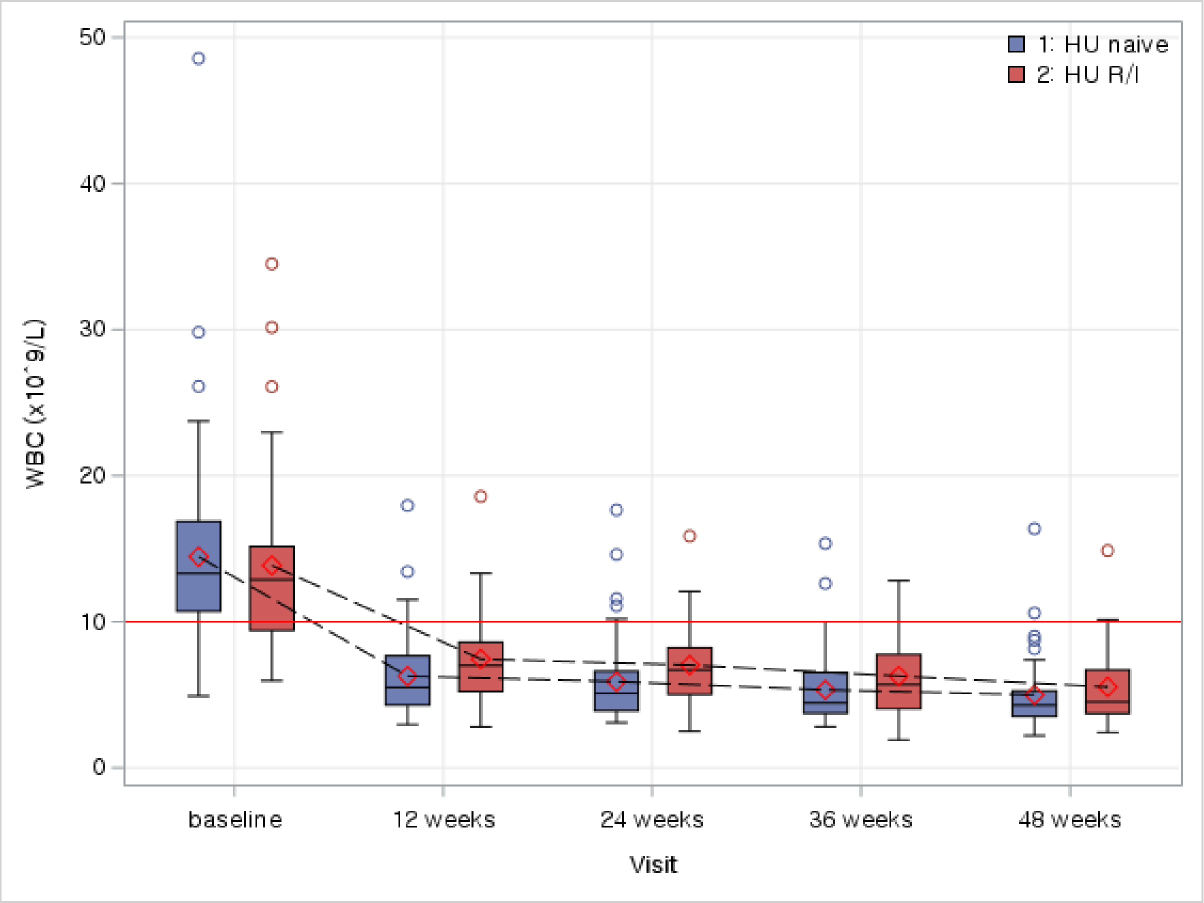

(C) Change platelet count ($10^9$/L)

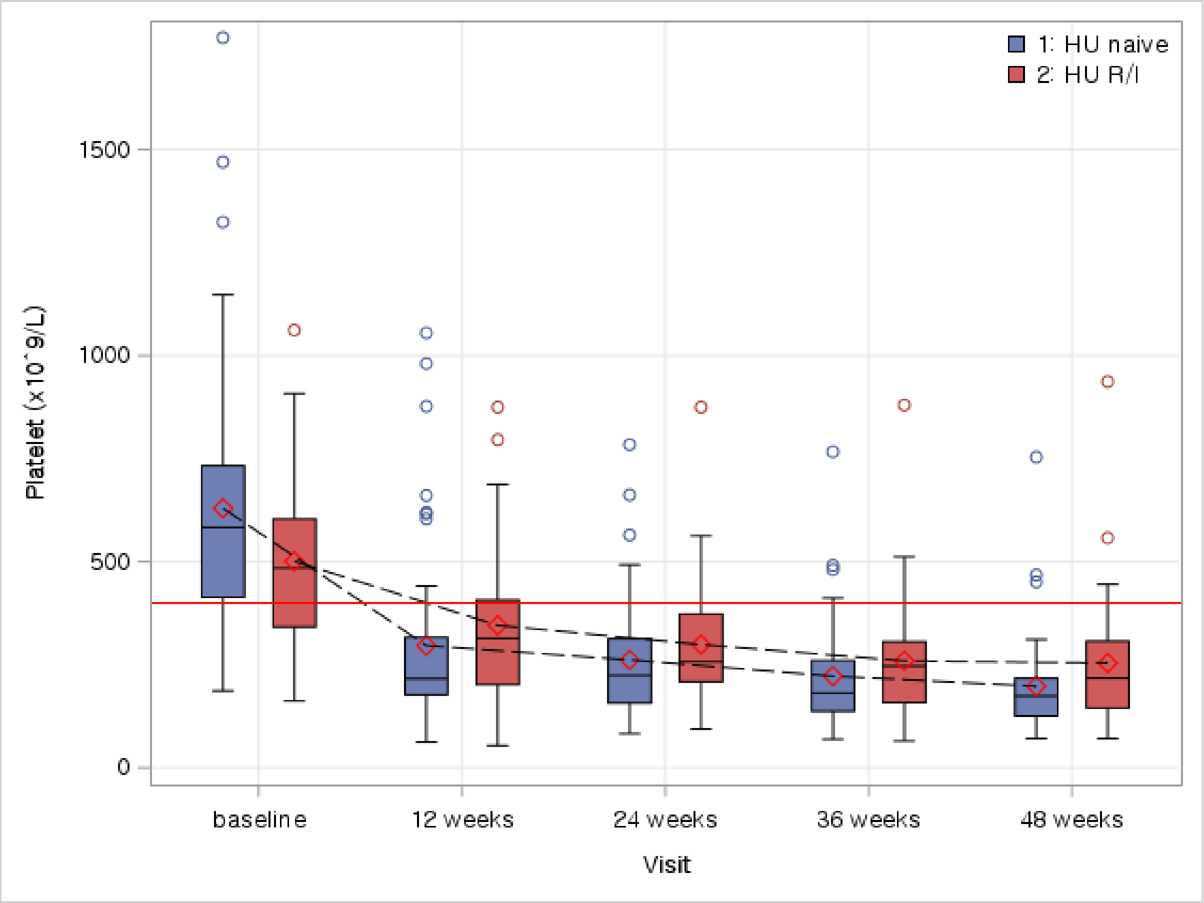

(D) % change of JAK2V617F allele burden (%)

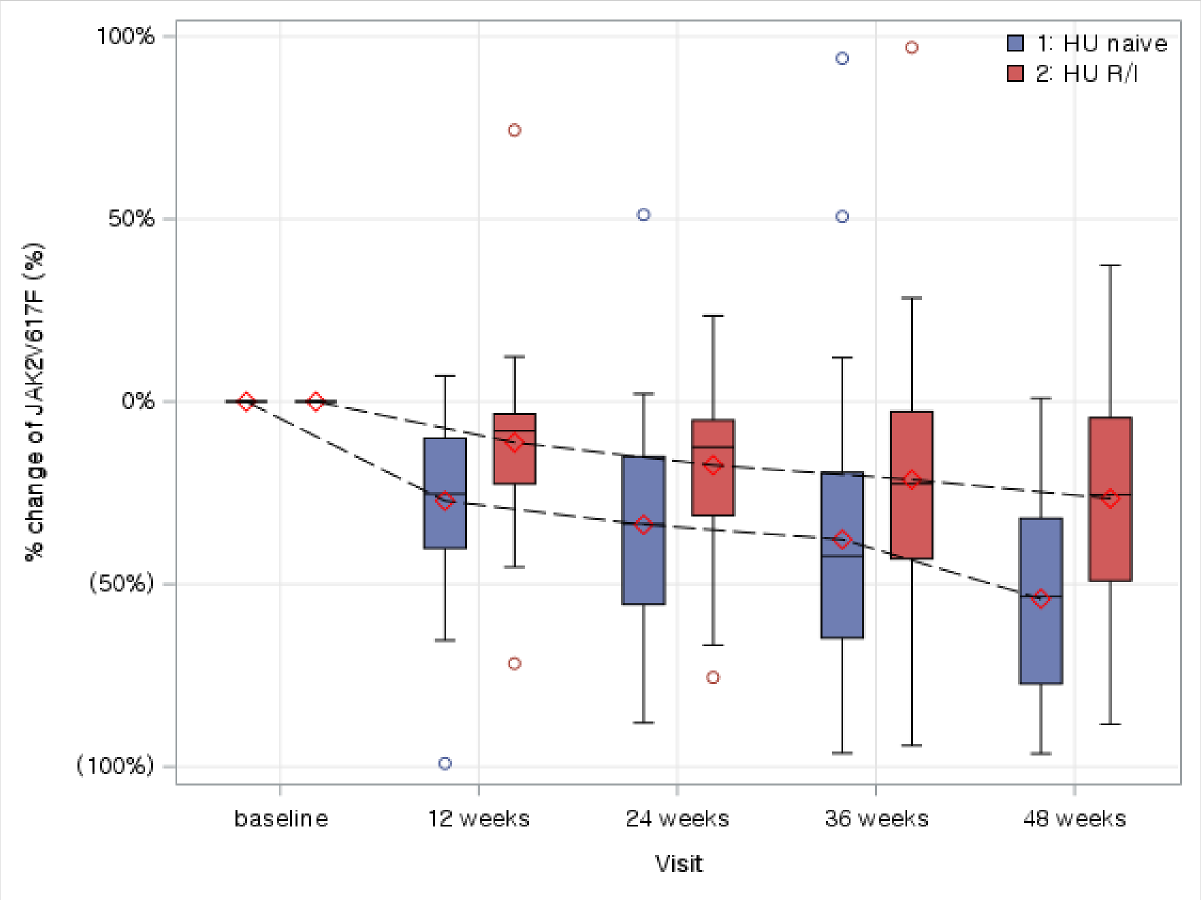

Figure S 5. Change of hematologic parameters ((A) Hct, (B) WBC, (C) Platelets) and (D) JAK2V617F allele burden during the core treatment period by Low-risk vs High-risk. The means of the plot are connected with straight lines, and the redline indicates baseline value for assessing CHR for each hematologic parameter (Hct 45%, WBC $10x10^9$/L, PLT $400x10^9$/L).

(A) Change hematocirt (%)

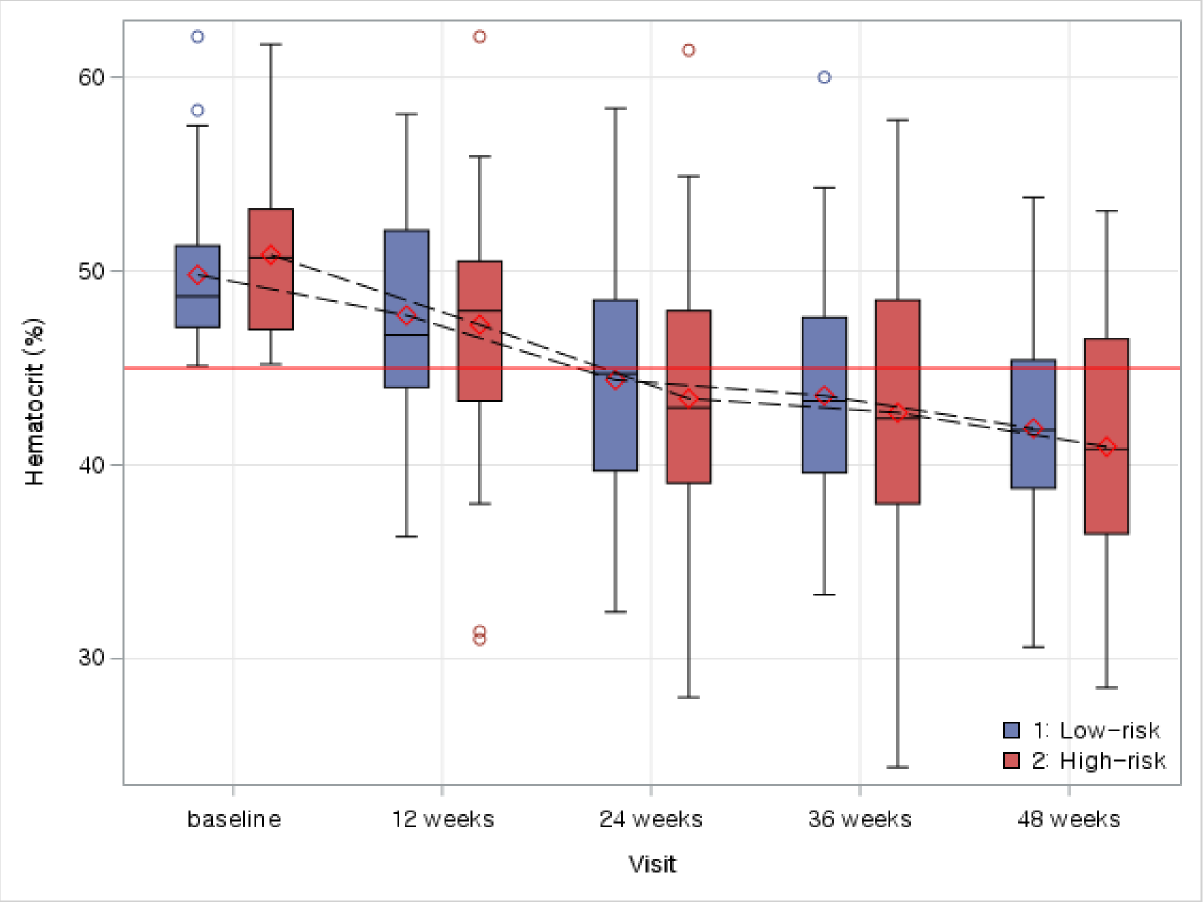

(B) Change white blood cell count ($10^9$/L)

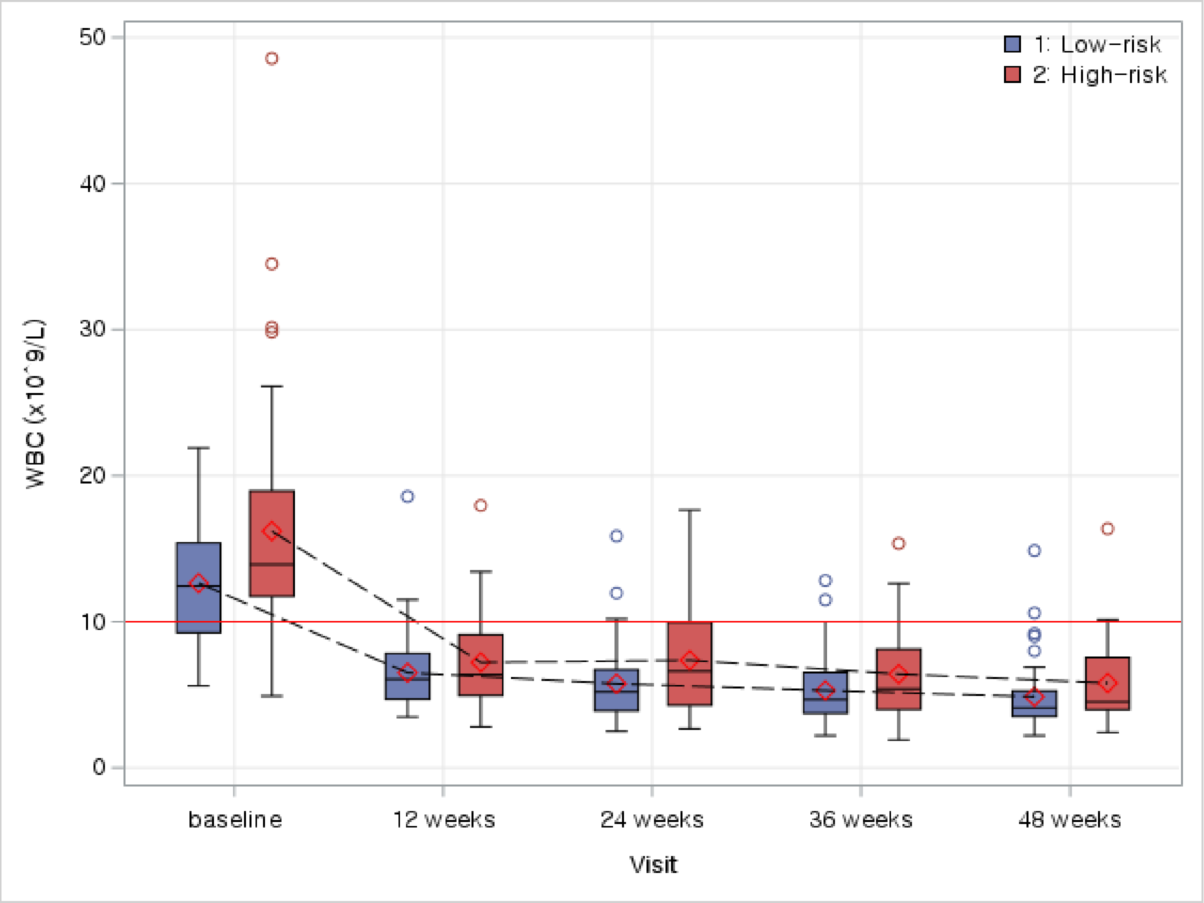

(C) Change platelet count ($10^9$/L)

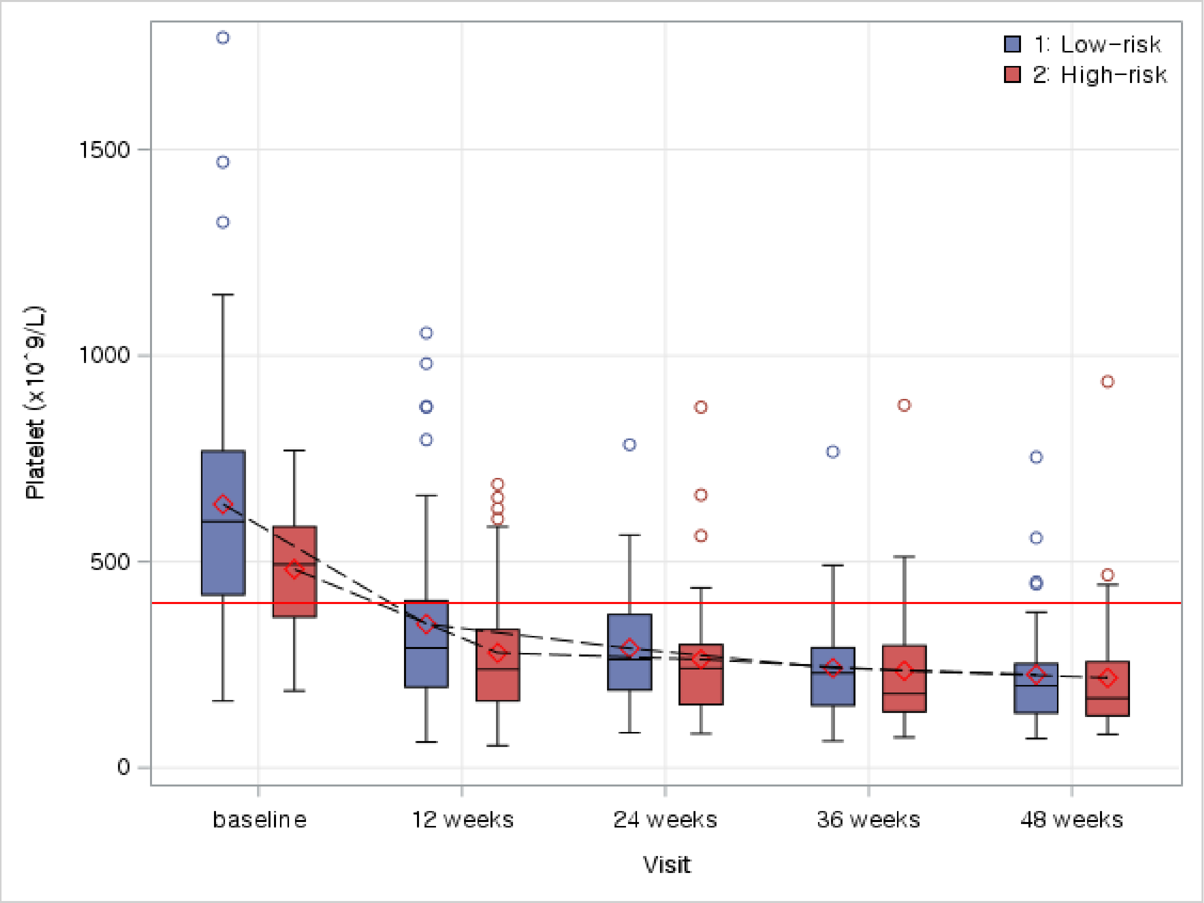

(D) % change of JAK2V617F allele burden (%)

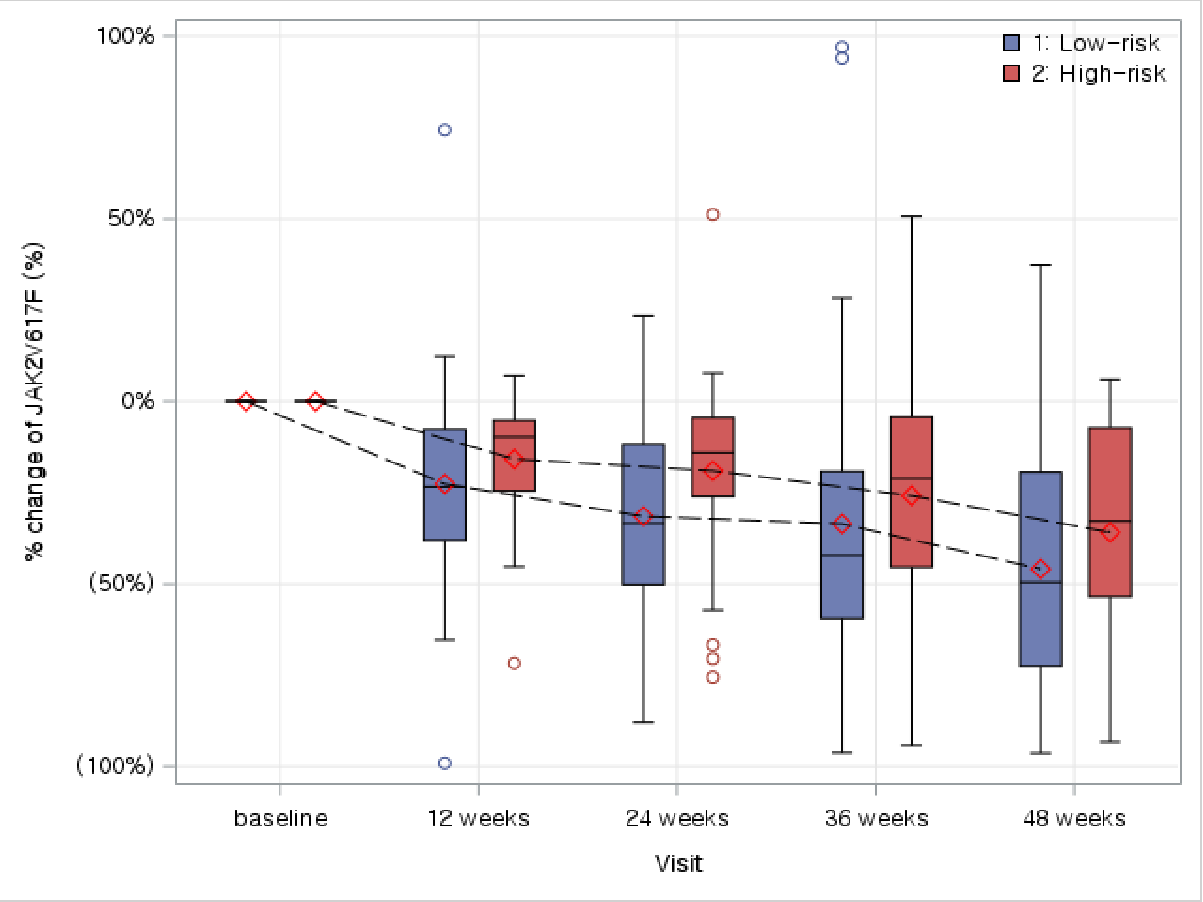


Figure S 6. Frequent drug-related AEs by treatment duration (according to investigators)

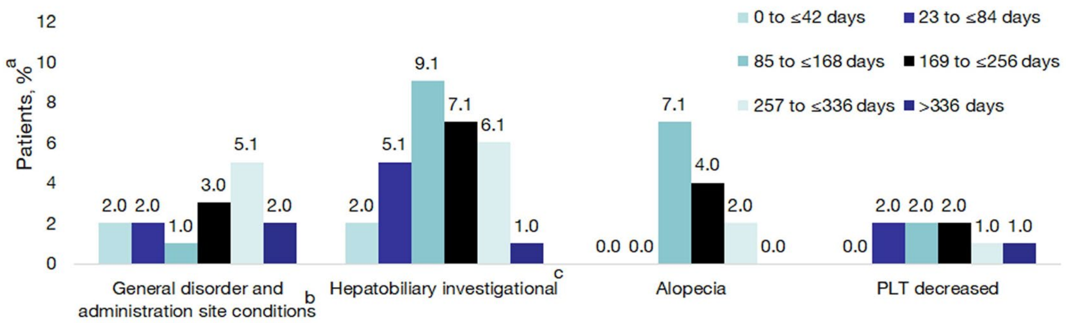


a. A patient with multiple occurrence of an adverse event is counted only once in that time period. The denominator for incidence is safety set (N=99)
b. General disorder and administration site conditions: Flu-like syndrome, fatigue, asthenia, pyrexia, injection site related, mucosal inflammation
c. Hepatobiliary investigational: AST/ALT, GGT, biliary enzyme, hepatic enzyme increased
